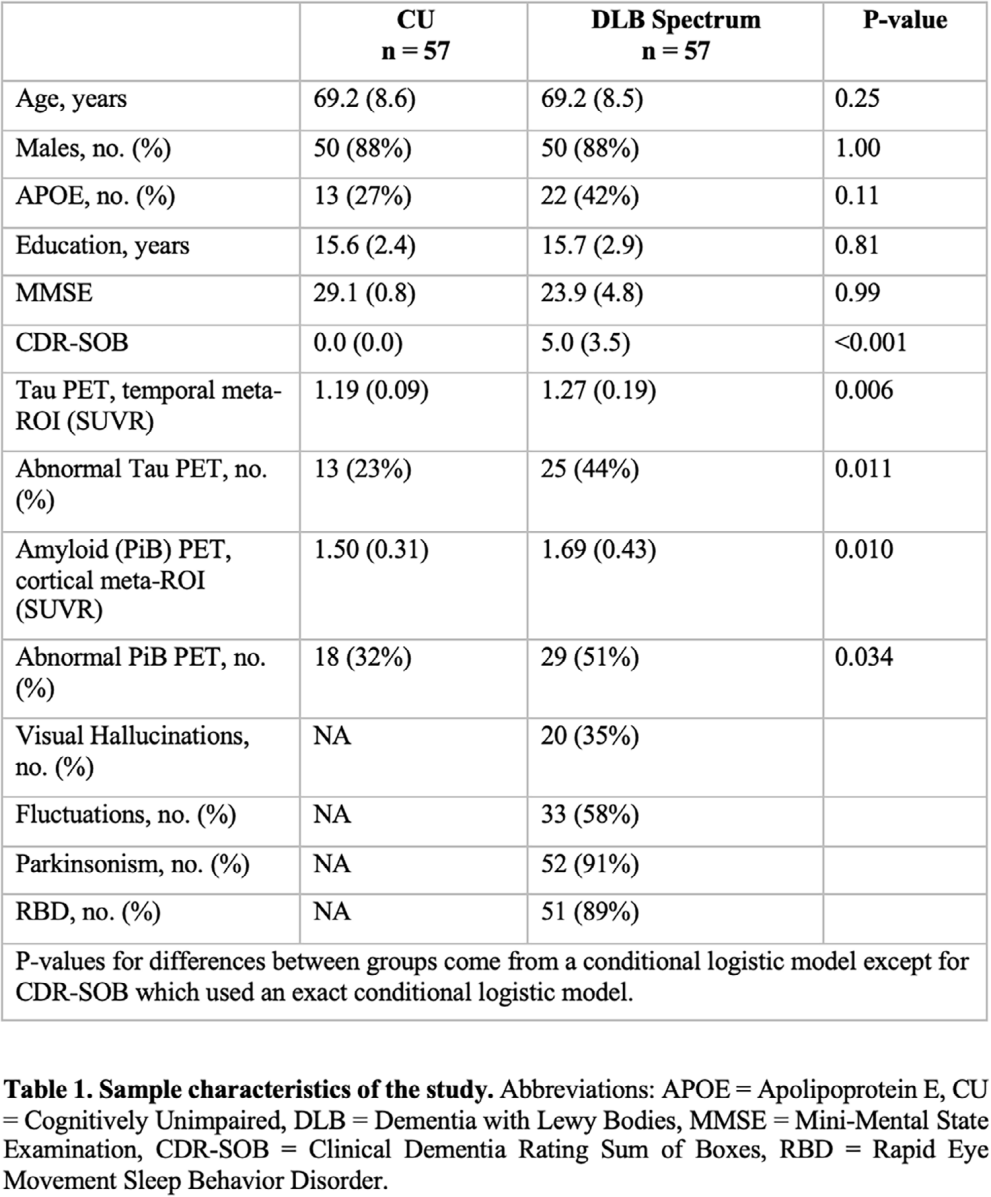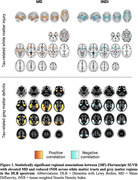# Tau deposition is associated with neurite abnormalities in white matter tracts and cortical grey matter regions in dementia with Lewy bodies

**DOI:** 10.1002/alz.089309

**Published:** 2025-01-09

**Authors:** Elijah Mak, Robert I. Reid, Scott A. Przybelski, Timothy G. Lesnick, Christopher G. Schwarz, Matthew L. Senjem, Sheelakumari Raghavan, Prashanthi Vemuri, Clifford R. Jack, Hoon‐Ki Min, Manoj K. Jain, Toji Miyagawa, Leah K. Forsberg, Julie A. Fields, Rodolfo Savica, Jonathan Graff‐Radford, David T. Jones, Hugo Botha, Erik K St. Louis, David S. Knopman, Vijay K. Ramanan, Dennis W. Dickson, Neill R Graff‐Radford, Tanis J Ferman, Ronald C. Petersen, Val J. Lowe, Brad Boeve, John T O'Brien, Kejal Kantarci

**Affiliations:** ^1^ Department of Psychiatry, University of Cambridge, Cambridge UK; ^2^ Mayo Clinic, Rochester, MN USA; ^3^ Department of Quantitative Health Sciences, Mayo Clinic, Rochester, MN USA; ^4^ Mayo Clinic, Quantitative Health Sciences, Rochester, MN USA; ^5^ Mayo Clinic, Radiology, Rochester, MN USA; ^6^ Department of Radiology, Mayo Clinic, Rochester, MN USA; ^7^ Mayo Clinic, Jacksonville, FL USA; ^8^ Department of Neurology, Mayo Clinic, Rochester, MN USA; ^9^ Department of Psychiatry and Psychology, Mayo Clinic, Rochester, MN USA; ^10^ Department of Neuroscience, Mayo Clinic, Jacksonville, FL USA; ^11^ Department of Neurology, Mayo Clinic, Jacksonville, FL USA; ^12^ Department of Psychiatry and Psychology, Mayo Clinic, Jacksonville, FL USA

## Abstract

**Background:**

Dementia with Lewy bodies (DLB) frequently co‐occurs with Alzheimer’s disease (AD) pathologies, exacerbating disease progression. Biophysical models of diffusion imaging data, such as Neurite Orientation Dispersion and Density Imaging (NODDI), may reveal novel insights into the neurobiological substrates of AD on cortical and white matter microstructural injury.

**Method:**

A cohort of 57 DLB patients on the DLB spectrum (33 clinically probable DLB and 14 prodromal DLB) and 57 cognitively unimpaired (CU) controls underwent NODDI and PET imaging with [^18^F]‐Flortaucipir and [^11^C]‐PiB (Table 1). NODDI parameters were derived from white matter tracts and grey matter regions using the JHU “Eve” and Mayo Clinic Adult Lifespan Template atlases, respectively. Conditional logistic models were used to compare these parameters between the two groups, accounting for the 1:1 matching on age and sex. The associations of tau and amyloid‐β with NODDI parameters across the white matter tracts were examined with linear regressions. A region‐by‐region approach was adopted to evaluate the relationships of tau and amyloid‐β with grey matter NODDI, adjusting for age, APOE, and local grey matter volumes.

**Result:**

DLB spectrum patients exhibited extensive white matter abnormalities relative to CU, including lower fractional anisotropy, higher mean diffusivity (MD), and lower tissue‐weighted neurite density (tNDI). In grey matter, these deficits were paralleled with higher MD and lower tNDI and ODI, primarily in the temporoparietal and posterior cortices. Regression models revealed significant associations of elevated [^18^F]‐Flortaucipir uptake with deficits in temporal and limbic white matter tracts and a temporal‐lobe pattern of higher MD and lower tNDI in the cortex (Figure 1). No associations were observed with [^11^C]‐PiB in the DLB spectrum.

**Conclusion:**

These findings demonstrated an *in vivo* relationship between tau deposition and neurite deficits that spatially aligned with the canonical AD “cortical signature” of neurodegeneration in both white matter tracts and grey matter regions in DLB. Our study underscores the detrimental impact of AD pathology in DLB and provides a rationale for further investigations into the underlying mechanisms of AD in DLB. Future longitudinal studies tracking the progression of these changes could inform therapeutic strategies for DLB in the presence of AD pathologies